# Reduction of Superoxide Dismutase 1 Delays Regeneration of Cardiotoxin-Injured Skeletal Muscle in KK/Ta-*Ins2^Akita^* Mice with Progressive Diabetic Nephropathy

**DOI:** 10.3390/ijms22115491

**Published:** 2021-05-23

**Authors:** Yuya Takahashi, Tatsunori Shimizu, Shunsuke Kato, Mitsuhiko Nara, Yumi Suganuma, Takehiro Sato, Tsukasa Morii, Yuichiro Yamada, Hiroki Fujita

**Affiliations:** 1 Department of Metabolism and Endocrinology, Akita University Graduate School of Medicine, 1-1-1 Hondo, Akita 010-8543, Japan; rounen-yuya@gipc.akita-u.ac.jp (Y.T.); nottomato2@yahoo.co.jp (T.S.); katoshun@gipc.akita-u.ac.jp (S.K.); mitsugoo.1037@gipc.akita-u.ac.jp (M.N.); y-suga@gipc.akita-u.ac.jp (Y.S.); takehiro@med.akita-u.ac.jp (T.S.); morii@gipc.akita-u.ac.jp (T.M.); yamada.yuuichiro@a3.kepco.co.jp (Y.Y.); 2 Kansai Electric Power Medical Research Institute, 2-1-7 Fukushima-ku, Osaka 553-0003, Japan

**Keywords:** akita mouse, cardiotoxin injury, diabetic nephropathy, muscle regeneration, oxidative stress, superoxide dismutase 1

## Abstract

Superoxide dismutase (SOD) is a major antioxidant enzyme for superoxide removal, and cytoplasmic SOD (SOD1) is expressed as a predominant isoform in all cells. We previously reported that renal SOD1 deficiency accelerates the progression of diabetic nephropathy (DN) via increasing renal oxidative stress. To evaluate whether the degree of SOD1 expression determines regeneration capacity and sarcopenic phenotypes of skeletal muscles under incipient and advanced DN conditions, we investigated the alterations of SOD1 expression, oxidative stress marker, inflammation, fibrosis, and regeneration capacity in cardiotoxin (CTX)-injured tibialis anterior (TA) muscles of two Akita diabetic mouse models with different susceptibility to DN, DN-resistant C57BL/6-*Ins2^Akita^* and DN-prone KK/Ta-*Ins2^Akita^* mice. Here, we report that KK/Ta-*Ins2^Akita^* mice, but not C57BL/6-*Ins2^Akita^* mice, exhibit delayed muscle regeneration after CTX injection, as demonstrated by the finding indicating significantly smaller average cross-sectional areas of regenerating TA muscle myofibers relative to KK/Ta-wild-type mice. Furthermore, we observed markedly reduced SOD1 expression in CTX-injected TA muscles of KK/Ta-*Ins2^Akita^* mice, but not C57BL/6-*Ins2^Akita^* mice, along with increased inflammatory cell infiltration, prominent fibrosis and superoxide overproduction. Our study provides the first evidence that SOD1 reduction and the following superoxide overproduction delay skeletal muscle regeneration through induction of overt inflammation and fibrosis in a mouse model of progressive DN.

## 1. Introduction

Sarcopenia is a muscle disease characterized by degenerative loss of skeletal muscle mass, leading to a reduction in muscle strength or physical function. Recently, sarcopenia has been recognized as a novel diabetic complication, most often seen in elderly diabetic patients (>65 years old) [1]. Notably, accumulating evidence suggests that muscle regeneration pathways are dysregulated in elderly adults with sarcopenia [2]. Moreover, chronic kidney disease (CKD) is known to be a critical risk factor for the development of sarcopenia. Indeed, clinical studies have indicated that patients with decreased glomerular filtration rate (GFR) exhibit a high frequency of muscle loss [3] and have diminished walking speed and decreased muscle strength [4]. Diabetic nephropathy (DN) is the leading cause of CKD worldwide, and therefore it may be a potential contributor to the development of sarcopenia. However, the mechanism underlying the relationship between sarcopenia and DN is poorly understood.

Oxidative stress induced by hyperglycemia is well known to be implicated in the development and progression of diabetic complications [5]. Under hyperglycemic condition, excessive superoxide anion is generated by multiple pathways including mitochondrial electron-transport chain, NAD(P)H oxidase, and uncoupled endothelial nitric oxide synthase [6,7], leading to the cell and tissue injury in various organs [8]. To protect organs from oxidative stress induced by superoxide excess, superoxide dismutase (SOD) works for superoxide removal in body [9]. SOD converts superoxide into hydrogen peroxide and molecular oxygen, and then the hydrogen peroxide is detoxified to water by catalase or glutathione peroxidase [10]. Thus, SOD plays a critical role in the antioxidant defense system and would serve as a defender against various diabetic complications, including sarcopenia.

*Ins2^Akita^* mouse (Akita mouse) is a mouse model of non-obese hypoinsulinemic diabetes. Through the analysis of two *Ins2^Akita^* diabetic mouse models showing different susceptibility to the development and progression of DN, DN-resistant C57BL/6-*Ins2^Akita^* (C57BL/6-Akita) and DN-prone KK/Ta-*Ins2^Akita^* (KK/Ta-Akita), we have demonstrated that cytoplasmic copper/zinc-SOD (SOD1) is down-regulated in the kidneys of KK/Ta-Akita mice that exhibit progressive DN, but not DN-resistant C57BL/6-Akita mice [11], and that genetic SOD1 deficiency accelerates diabetic glomerular injury in the DN-resistant C57BL/6-Akita mice [12]. Additionally, we have verified that the impairment of antioxidant defense system by renal SOD1 down-regulation and deficiency increases renal oxidative stress in the setting of diabetes and triggers overt renal injury [12].

Given these lines of evidence indicating the protective role of SOD1 against renal oxidative damage, we hypothesized that the degree of SOD1 expression in skeletal muscle may be involved in muscle regeneration competence, which determines sarcopenic skeletal muscle phenotypes under hyperglycemic condition, and that the muscle SOD1 may be regulated in a DN severity-dependent manner. The aim of the present study was to test this hypothesis. For this purpose, we investigated the alterations of SOD1 expression, oxidative stress marker, inflammation, fibrosis, and regeneration capacity in cardiotoxin (CTX)-injured tibialis anterior (TA) muscles of two Akita diabetic mouse models with different susceptibility to DN, DN-resistant C57BL/6-Akita and DN-prone KK/Ta-Akita mice, and analyzed the role of SOD1 in regeneration of injured muscle in advanced DN.

## 2. Results

### 2.1. Physiological and Biochemical Parameters in C57BL/6-Akita and KK/Ta-Akita Mice at 15 Weeks of Age

In the present study, we used two Akita diabetic mouse models that are different regarding strain and susceptibility to DN, DN-resistant C57BL/6-Akita and DN-prone KK/Ta-Akita mice. Table 1 shows physiological and biochemical parameters in 15-week-old male C57BL/6-Akita and KK/Ta-Akita mice. C57BL/6-Akita and KK/Ta-Akita mice exhibited comparable values regarding body weight, systolic blood pressure, and blood glucose. Plasma creatinine, blood urea nitrogen, and total cholesterol were significantly higher in KK/Ta-Akita mice than in KK/Ta-wild-type (KK/Ta-WT) mice. Comparing the two Akita mice, blood urea nitrogen, total cholesterol, and triglyceride were significantly elevated in KK/Ta-Akita mice as compared with C57BL/6-Akita mice. Urinary albumin levels in KK/Ta-Akita mice were markedly increased relative to KK/Ta-WT mice. Despite comparable blood glucose levels between the two Akita mice, urinary albumin levels in C57BL/6-Akita mice were not significantly higher than those in C57BL/6-WT mice. Expectedly, grip strength values were lower in the two Akita mice than their WT mice in the same strain. Interestingly, grip strength in DN-prone KK/Ta-Akita mice was significantly reduced relative to DN-resistant C57BL/6-Akita mice.

### 2.2. Renal Phenotype in C57BL/6-Akita and KK/Ta-Akita Mice at 15 Weeks of Age

**Figure 1:**
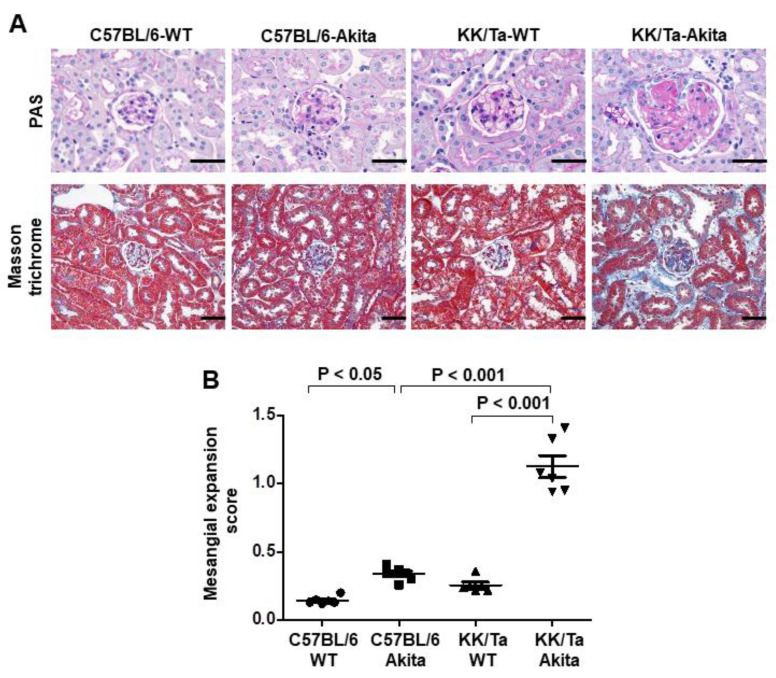
Renal histopathology in C57BL/6-Akita and KK/Ta-Akita mice. (**A**) Representative renal cortical images of PAS and Masson trichrome staining in C57BL/6-WT, C57BL/6-Akita, KK/Ta-WT, and KK/Ta-Akita mice at 15 weeks of age. Bars = 50 μm. (**B**) Glomerular mesangial expansion scores. Data are presented as means ± SEM. *n* = 6 per group.

Consistent with the findings from our previous reports [11,13], KK/Ta-Akita mice exhibited overt glomerular mesangial expansion and nodular lesions, as evidenced by increased accumulation of periodic acid-Schiff (PAS)-positive material in the mesangial area, and also moderate renal interstitial fibrosis, as shown in the Masson trichrome staining image (Figure 1A). In contrast, glomerular mesangial expansion and renal interstitial fibrosis were relatively mild in C57BL/6-Akita mice (Figure 1A). Semiquantitative analysis of PAS-stained kidney sections indicated significantly higher mesangial expansion score in KK/Ta-Akita mice relative to KK/Ta-WT and C57BL/6-Akita mice (Figure 1B).

### 2.3. Inflammation and Fibrosis in TA Muscles Following CTX Injection-Induced Injury

**Figure 2:**
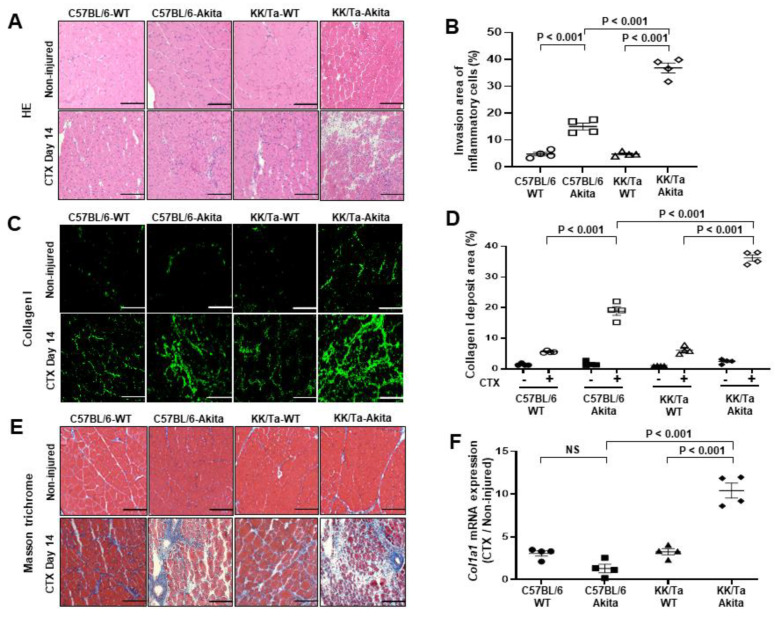
Inflammatory and fibrotic changes in TA muscles on day 14 after saline (non-injured) or CTX injection. (**A**) Representative images of HE staining in TA muscles. (**B**) Invasion area of HE-stained inflammatory cells in TA muscles on day 14 after CTX injection. (**C**) Representative images of collagen I immunofluorescent staining in TA muscles. (**D**) Analysis of collagen I deposit area in collagen I-stained images in TA muscles on day 14 after CTX injection. CTX − and CTX + indicate saline and CTX injection, respectively. (**E**) Representative images of Masson trichrome staining in TA muscles. (**F**) *Col1a1* mRNA expression analysis in TA muscles on day 14 after CTX injection. Bars = 200 μm for all images. Data are presented as means ± SEM. *n* = 4 per group.

CTX injection is one of the most frequently used methods to experimentally induce muscle injury by causing myolysis of myofibers [14,15,16,17]. On day 14 after CTX injection, examination of hematoxylin-eosin (HE)-stained muscle tissue sections revealed that markedly increased inflammatory cell infiltration was observed in TA muscle interstitial spaces of KK/Ta-Akita mice, while the cell infiltration was mild in those of C57BL/6-Akita mice (Figure 2A,B). Collagen I as an extracellular matrix is known to be increased in muscle degeneration induced by aging and CTX chemical injury [18,19,20]. In the current study, collagen I deposition was markedly increased in TA muscles of KK/Ta-Akita mice on day 14 after CTX injection relative to those of C57BL/6-Akita mice (Figure 2C,D). Additionally, the analysis of Masson trichrome-stained muscle sections indicated prominent fibrotic changes in TA muscles of KK/Ta-Akita mice on day 14 after CTX injection (Figure 2E). The mRNA expression analysis of *Col1a1*, which provides instructions for making collagen I, revealed that *Col1a1* expression levels were markedly elevated in CTX-injured TA muscles of KK/Ta-Akita mice as compared with those of C57BL/6-Akita and KK/Ta-WT mice (Figure 2F).

### 2.4. Regeneration of TA Muscles Following CTX Injection-Induced Injury

**Figure 3:**
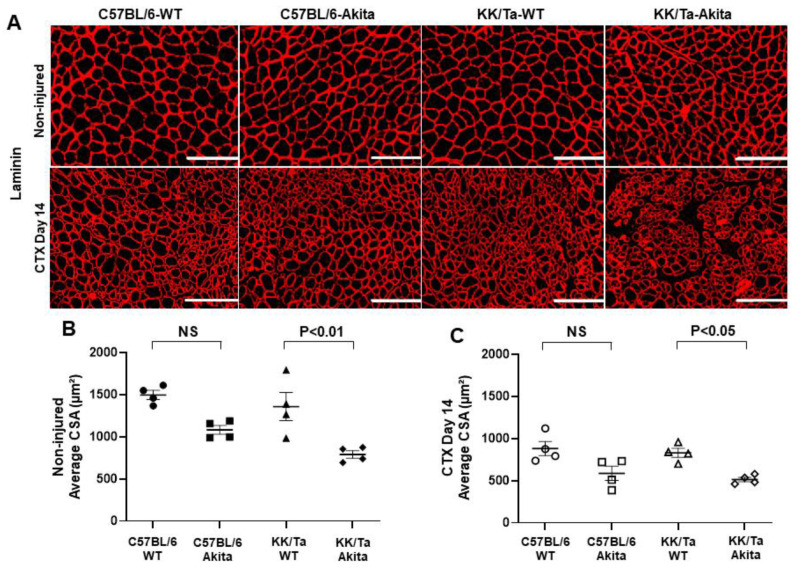
Assessment of regeneration in TA muscles on day 14 after saline (non-injured) or CTX injection. (**A**) Representative images of laminin immunofluorescent staining in TA muscles. Bars = 200 μm. (**B**) Average cross-sectional area (CSA) of regenerating TA muscle myofibers on day 14 after saline injection. (**C**) Average CSA of regenerating TA muscle myofibers on day 14 after CTX injection. Data are presented as means ± SEM. *n* = 4 per group.

To evaluate the regeneration competence of CTX-injured TA muscles in C57BL/6-Akita and KK/Ta-Akita mice, we analyzed the myofiber cross-sectional area in laminin-stained TA muscle sections. Through the morphometrical analysis of myofiber, KK/Ta-Akita mice exhibited significantly smaller average cross-sectional areas of myofibers in saline-injected TA muscles as compared with KK/Ta-WT mice (Figure 3A,B). Additionally, on day 14 after CTX injection, KK/Ta-Akita mice showed significantly smaller average cross-sectional areas of regenerating TA muscle myofibers, indicating delayed muscle regeneration relative to KK/Ta-WT mice (Figure 3A,C). In contrast, the average cross-sectional areas of myofibers were not significantly different between C57BL/6-WT and C57BL/6-Akita mice on day 14 after both saline and CTX injections (Figure 3A–C).

### 2.5. Changes in Oxidative Stress and SOD1 Expression in TA Muscles Following CTX Injection-Induced Injury

**Figure 4:**
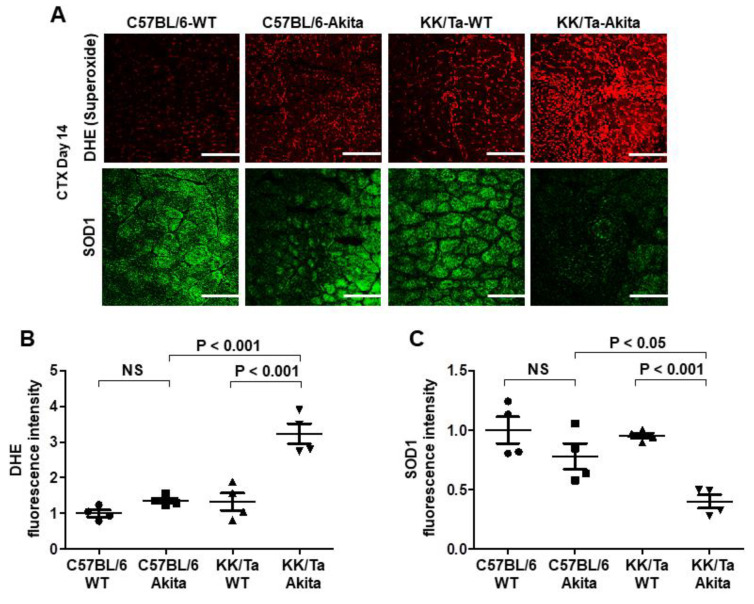
Changes in superoxide production and SOD1 expression in TA muscles on day 14 after CTX injection. (**A**) Representative images of DHE and SOD1 immunofluorescent stainings in TA muscles on day 14 after CTX injection. The degree of superoxide production was evaluated by DHE fluorescence intensity. Bars = 200 μm. (**B**) The average semiquantified fluorescence intensity of DHE staining. DHE fluorescence intensity was assessed in 6 TA muscle regions per mouse, and the average value was calculated in each mouse. (**C**) The average semiquantified fluorescence intensity of SOD1 immunofluorescent staining. SOD1 fluorescence intensity was assessed in 6 TA muscle regions per mouse, and the average value was calculated in each mouse. Data are presented as means ± SEM. *n* = 4 per group.

Muscle superoxide levels were assessed by dihydroethidium (DHE) histochemistry. On day 14 after CTX injection, superoxide production was increased in TA muscles of KK/Ta-Akita mice as compared with C57BL/6-Akita and KK/Ta-WT mice, indicating increased oxidative stress within TA muscles of KK/Ta-Akita mice during the muscle regeneration process (Figure 4A,B). Notably, SOD1, which is a major antioxidant defender against superoxide and oxidative stress, was markedly down-regulated in TA muscles of KK/Ta-Akita mice relative to C57BL/6-Akita and KK/Ta-WT mice on day 14 after CTX injection (Figure 4A,C). These data suggest that DN-prone KK/Ta-Akita mice have increased superoxide production and impaired antioxidant defense capacity not only in their kidneys, but also in their muscles.

## 3. Discussion

To explore the mechanism underlying the relationship between sarcopenia and DN, we used two Akita diabetic mouse models with different susceptibility to DN, DN-resistant C57BL/6-Akita and DN-prone KK/Ta-Akita mice in the present study. KK/Ta-Akita mice are characterized by overt albuminuria and prominent diabetic glomerulosclerosis, as previously reported [11]. In contrast, C57BL/6-Akita mice are known to develop only mild albuminuria and minor glomerular lesions [11]. Additionally, in this study, such renal phenotypes were observed in the two Akita diabetic mouse models (Table 1; Figure 1).

Regarding physiological parameter of sarcopenia, KK/Ta-Akita mice with progressive DN exhibited lower grip strengths as compared with DN-resistant C57BL/6-Akita mice (Table 1). This finding suggests a potential relationship between sarcopenia and the severity of DN. The development of sarcopenia is known to rely mainly on dysregulation of muscle regeneration [2,21]. Additionally, accumulating evidence suggests that inflammation and fibrosis influence the muscle regeneration process [22]. Given these lines of evidence, it is conceivable that the inflammatory and fibrotic changes in skeletal muscle may be induced under pathological environments such as progressive DN, and further accelerated by exposure to various stimuli including chemicals or by aging. Therefore, we first focused on the investigation of inflammation and fibrosis in the skeletal muscle regeneration process following CTX injection, which is a widely used method to experimentally induce muscle injury. Our results demonstrate that inflammatory and fibrotic changes are highly caused during the regeneration process of chemically injured muscle under progressive DN conditions, as evidenced by excessive inflammatory cell infiltration, enhanced collagen I deposition, and increased expression of *Col1a1* mRNA, which is responsible for making collagen I in skeletal muscles of the DN-prone KK/Ta-Akita mice (Figure 2). Collagen is a major component of extracellular matrix (ECM), and it has an essential role in muscle regeneration [22]. However, excessive collagen accumulation, which is defined as fibrosis, would adversely affect muscle regeneration after injury [23]. In this regard, Hu et al. reported impaired regeneration of myofibers by excessive muscle collagen deposition after CTX injury in high-fat diet-induced mice [24]. Moreover, Krause et al. reported that an impairment in collagen remodeling after intramuscular CTX injection delayed muscle regeneration in C57BL/6-Akita mice [20].

Generally, skeletal muscles have a tremendous capacity for repair and regeneration in response to injury. However, this process can be compromised in several pathological conditions such as myopathies, muscular dystrophy and sarcopenia [14,17,25]. Considering these characteristics of skeletal muscle, we next investigated the degrees of muscle regeneration following CTX injury through laminin immunohistochemical analysis and compared them between the two Akita diabetic mouse models. It is noteworthy that KK/Ta-Akita mice with progressive DN, but not DN-resistant C57BL/6-Akita mice, showed significantly smaller average cross-sectional areas of myofibers relative to their WT controls, KK/Ta-WT mice, in both CTX-injected and saline-injected TA muscles. These findings indicate that muscle regeneration capacity may depend on the severity of DN. Indeed, several experimental studies on CTX-injured muscles have shown a reduced muscle regeneration competence in mouse models of subtotally nephrectomized mice with chronic kidney disease [26] and obese ob/ob and db/db mice with overt albuminuria [27].

Oxidative stress has been shown to be a crucial factor that affects the muscle regeneration process [28,29]. To mitigate oxidative damage, SOD works as a defender against oxidative stress in body. In mammals, there are three SOD isoforms of cytoplasmic SOD1, mitochondrial manganese-SOD (SOD2), and extracellular copper/zinc-SOD (SOD3) [10,30]. Of the three isoforms, SOD1 is considered to be a major isoform, since it is expressed as a predominant isoform in all cells. Additionally, it is known that SOD1 accounts for 50% to 80% of total SOD activity, SOD2 accounts for 2% to 12%, and SOD3 accounts for the remainder in normal mouse arteries [10,31]. Furthermore, our previous studies have verified that SOD1 deficiency causes overt diabetic renal injury in the DN-resistant C57BL/6-Akita mice [12], and that SOD1 expression is down-regulated in the kidneys of DN-prone KK/Ta-Akita mice [11]. Based on these lines of evidence, we speculated that antioxidative defense capacity by SOD1 may also be reduced in the skeletal muscles of DN-prone KK/Ta-Akita mice. As expected, our results clearly indicate down-regulated SOD1 expression and increased oxidative stress in the skeletal muscles of DN-prone KK/Ta-Akita mice after CTX injection (Figure 4). In addition, we showed the notable findings that SOD1 expression is relatively maintained and therefore oxidative stress is not significantly increased in the skeletal muscles of DN-resistant C57BL/6-Akita mice relative to their WT controls even after CTX injection (Figure 4). Similar to our findings, a recent experimental study has indicated that increased oxidative stress and decreased SOD1 expression are observed in skeletal muscles of 5/6 nephrectomized rats as a CKD model [32]. Taken together, the current findings suggest critical roles of SOD1 in protection against muscle oxidative stress and in muscle regeneration, and a close relationship between muscle SOD1 reduction and DN severity or the degree of CKD.

Satellite cells are well known to play an important role in muscle regeneration [33,34]. When skeletal muscle is injured, quiescent satellite cells are activated. The activated satellite cells undergo active proliferation, and they contribute to differentiation of myoblasts and regeneration of myofibers [35]. Hence, it appears to be plausible that the satellite cells are negatively regulated in progressive DN, resulting in delayed muscle regeneration. Unfortunately, the current study has not clarified the alterations of satellite cell activation and proliferation during muscle injury in advanced DN. Further study would be required about this point.

**Figure 5:**
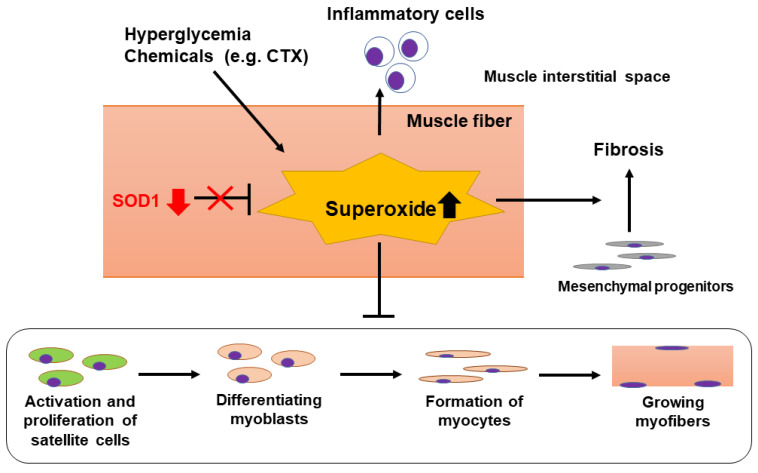
Proposed mechanism of delayed skeletal muscle regeneration in DN-prone KK/Ta-Akita mice. During exposure to hyperglycemia or chemicals such as CTX, excessive superoxide anions are produced in muscle fibers. Oxidative stress induced by superoxide overproduction would increase inflammatory cell infiltration into the muscle interstitial spaces, accelerate muscle interstitial fibrosis, and delay differentiation of myoblasts and regeneration of myofibers via suppressing activation and proliferation of satellite cells. When SOD1 removes superoxide anions effectively, these inflammatory and fibrotic changes would be attenuated, leading to promoting regeneration of injured muscles. Hence, SOD1 down-regulation observed in TA muscles of DN-prone KK/Ta-Akita mice following CTX injection may be involved in their delayed skeletal muscle regeneration.

A possible mechanism of delayed skeletal muscle regeneration in progressive DN is summarized in Figure 5. The present study provides the first evidence that SOD1 reduction and the following superoxide overproduction delay skeletal muscle regeneration through induction of overt inflammation and fibrosis in advanced DN. However, further studies are needed to clarify a more detailed mechanism underlying the regulation of muscle regeneration via the signaling from satellite cells in DN. Finally, the current results would give a new insight into the pathogenesis of sarcopenia in DN and contribute to the development of better treatment strategies for this disease.

## 4. Materials and Methods

### 4.1. Experimental Animals

C57BL/6-Akita mice are available from SLC (Hamamatsu, Shizuoka, Japan). KK/Ta-Akita mice were generated in our laboratory as described previously [11]. In the present study, 12- to 15-week-old male C57BL/6-WT, C57BL/6-Akita, KK/Ta-WT, and KK/Ta-Akita mice were used for the histopathologic assessment of renal tissues and for the histological evaluation of skeletal muscle tissues following cardiotoxin injury. The mice were allowed unrestricted access to standard rodent chow and water. The animal work was reviewed and approved by the Committee on Animal Experimentation of Akita University (protocol number a-1-0245, approved 5 August 2020).

### 4.2. Measurements of Physiological and Biochemical Parameters

Blood glucose levels on samples obtained after a 6 h daytime fast were determined using Glutestmint (Sanwa Chemistry, Aichi, Japan). We measured plasma creatinine, blood urea nitrogen, plasma total cholesterol, and plasma triglyceride levels by using an autoanalyzer (Fuji Dry-Chem 5500, Fuji Film, Tokyo, Japan). Urinary albumin and creatinine concentrations on morning spot urine were measured by an Albuwell-M Murine Microalbuminuria ELISA kit (Exocell, Philadelphia, PA, USA) and Creatinine Companion kit (Exocell), respectively, and the urinary albumin-to-creatinine ratio was determined as described previously [36]. Systolic blood pressure and grip strength were determined using a non-invasive tail cuff and pulse transducer system (BP-98A, Softron, Tokyo, Japan) and a grip strength meter (MK-380Si, Muromachi Kikai, Tokyo, Japan), respectively. The measurements of grip strength were repeated ten times, and the maximum value of grip strength of each mouse is presented.

### 4.3. Renal Histology

We evaluated renal histopathology in male C57BL/6-WT, C57BL/6-Akita, KK/Ta-WT, and KK/Ta-Akita mice at 15 weeks of age. The removed kidneys were fixed in 4% paraformaldehyde in PBS overnight at 4 °C. Paraffin-embedded kidney tissues were cut to 2 μm thickness and then stained with periodic acid-Schiff (PAS) and Masson trichrome. The assessment of glomerular mesangial expansion was carried out using a semiquantitative score, as described previously [11].

### 4.4. Studies of Muscle Regeneration Following CTX Injury

Twelve-week-old male C57BL/6-WT, C57BL/6-Akita, KK/Ta-WT, and KK/Ta-Akita mice were used for the muscle regeneration studies. To prepare chemically injured muscle models, 100 μL of 10 μM cardiotoxin solution in saline was injected into left TA muscles. The right TA muscles were injected with the same volume of saline and served as the non-injured control. On day 14 after CTX injection, the mice were sacrificed, and TA muscles were removed and rapidly frozen. The muscles were cut at a position 2.5 mm from the proximal side, and 10 μm thick muscle tissue sections were prepared for HE staining, Masson trichrome staining, immunofluorescence histochemistry, and DHE histochemistry. The immunofluorescence histochemistry for collagen I, laminin, and SOD1 was performed using rabbit anti-collagen I (1:250; abcam, Cambridge, UK), rat anti-laminin (1:400; Santa Cruz, Dallas, TX, USA), and rabbit anti-Cu/Zn SOD (SOD1) (1:100; Stressgen, Ann Arbor, MI, USA) antibodies, respectively. The muscle superoxide levels were determined by DHE histochemistry, as described previously [11]. The fluorescent images were observed using confocal laser microscopy (LSM510; Carl Zeiss, Jena, Germany) or inverted fluorescence microscope (BZ-9000; Keyence, Osaka, Japan). The collagen Ⅰ accumulation area in muscle tissue sections was calculated using BZ-II analyzer software version 2.2 (Keyence). The myofiber cross-sectional areas in immunofluorescent-stained sections of laminin were measured in micrometers squared using BZ-II analyzer software version 2.2 (Keyence). The fluorescence intensity of DHE and SOD1 was semiquantified using Adobe Photoshop (version CS5; Adobe systems, San Jose, CA, USA).

### 4.5. Col1a1 mRNA Expression Analysis

Mouse total RNA was extracted from TA muscles using an RNeasy Mini Kit (Qiagen, Hilden, Germany), and then complementary DNA was synthesized using a Prime Script First-Strand cDNA Synthesis Kit (Takara, Shiga, Japan). Quantitative real-time PCR was performed using a Thermal Cycler Dice Real Time System (Takara) and SYBR Premix Ex tag II (Takara). Primer sequences were as follows: 5′-CATTGTGTATGCAGCTGACTTC-3′ (forward) and 5′-CGCAAAGAGTCTACATGTCTAG-3′ (reverse) for Col1a1; and 5′-CTCAACACGGGAAACCTCAC-3′ (forward) and 5′-GCTCCACCAACTAAGAACG-3′ (reverse) for 18S ribosomal RNA. The relative expression of Col1a1 transcripts was calculated as a ratio to 18S, and then the ratio of Col1a1 mRNA expression level in CTX-injured TA muscle to that in non-injured TA muscle was determined in each mouse.

### 4.6. Statistical Analysis

All data are presented as means ± SEM. Statistical analysis was performed using GraphPad Prism software (GraphPad, San Diego, CA, USA). Differences between multiple groups were determined by one-way ANOVA followed by Bonferroni’s multiple comparison test. *p*-value < 0.05 was considered statistically significant.

## Figures and Tables

**Table 1:** Physiological and biochemical parameters in 15-week-old C57BL/6-Akita and KK/Ta-Akita mice. Values are means ± SEM. *n* = 6 per each group except grip strength. *n* = 4 per each group for grip strength. * *p* < 0.05 vs. C57BL/6-WT. ^†^ *p* < 0.05 vs. C57BL/6-Akita. ^§^ *p* < 0.05 vs. KK/Ta-WT.

Parameter	C57BL/6-WT	C57BL/6-Akita	KK/Ta-WT	KK/Ta-Akita
Body weight (g)	23.4 ± 0.4	21.7 ± 0.3	29.8 ± 0.9	21.6 ± 0.4 ^§^
Systolic blood pressure (mmHg)	102 ± 5	117 ± 5 *	106 ± 3	116 ± 3 ^§^
Blood glucose (mmol/L)	9.5 ± 0.3	21.4 ± 2.9 *	9.4 ± 0.6	25.9 ± 1.8 ^§^
Plasma creatinine (µmol/L)	25.0 ± 2.7	22.1 ± 4.4	14.8 ± 2.9	35.4 ± 7.6 ^§^
Blood urea nitrogen (mmol/L)	9.0 ± 0.4	8.0 ± 1.1	8.3 ± 0.4	16.5 ± 2.1 ^†,§^
Total cholesterol (mmol/L)	1.79 ± 0.06	2.07 ± 0.30	1.74 ± 0.03	4.13 ± 0.23 ^†,§^
Triglyceride (mmol/L)	1.37 ± 0.12	1.17 ± 0.14	1.63 ± 0.09	2.37 ± 0.35 ^†^
Urinary albumin (µg/mg creatinine)	66 ± 25	141 ± 19	76 ± 18	631 ± 108 ^§^
Grip strength (kg)	0.272 ± 0.003	0.235 ± 0.003 *	0.227 ± 0.008	0.168 ± 0.004 ^†,§^

## Data Availability

Data are available upon reasonable request.
